# Experiences of Dietetic Students Taking Part in an Online Longitudinal Dementia Educational Programme: A Qualitative Study

**DOI:** 10.1111/jhn.70069

**Published:** 2025-05-28

**Authors:** Santiago Rodriguez Pena, Molly Hebditch, Stephanie Daley, Emily Norris, Cecile Jones, Sube Banerjee, Yvonne Feeney

**Affiliations:** ^1^ Brighton and Sussex Medical School, Department of Neuroscience University of Sussex Falmer Brighton UK; ^2^ School of Biosciences Guildford Surrey University of Surrey Surrey UK; ^3^ Teesside University School of Health and Life Sciences Middlesbrough Tees Valley UK; ^4^ Faculty of Medicine and Health Sciences, Medical School, Queens Medical Centre University of Nottingham Nottingham UK

**Keywords:** dementia, dietetic, education, longitudinal, online learning, undergraduate

## Abstract

**Introduction:**

Dietitians play a key role in the care of people with dementia, yet there is a lack of literature on the education of dietitians in this area. Dietetic students at the University of Surrey undertook the Time for Dementia programme as a mandatory component of their training to improve their knowledge of, and attitudes towards people with dementia. The programme was delivered online due to COVID‐19 restrictions. Students met with people with dementia and their carers over a 2‐year period. This study aimed to understand the learning experiences of dietetic students in Time for Dementia, and their perceptions of online delivery.

**Methods:**

All dietetic students undertaking the Time for Dementia educational programme during 2020 and 2021 were invited to take part in a qualitative study. Three focus groups were completed with 14 students on completion of a 2 year Time for Dementia programme. A semi‐structured topic guide was used to explore students' self‐reported learning outcomes, their experiences of online learning, and their learning experiences around dementia. The data was analysed using reflexive thematic analysis.

**Results:**

Three main themes were identified from the data. First, *gaining a holistic understanding of the experience of life with dementia*, described student learning on the lived experiences and the challenges faced by people with dementia. Second, *shaping future practice, adapting approaches for dementia care*, concerned the skills and attitudes that students highlighted as valuable for their future careers, and third, *optimising learning in an online environment*, outlined student's views and recommendations on online dementia education.

**Conclusions:**

Longitudinal contact with people with dementia can improve knowledge about the realities of life with dementia and inform future practice. The positive learning outcomes reported in this study suggest that online contact with families with dementia can be successfully incorporated into the dietetic undergraduate curriculum.

## Summary

- The Time for Dementia programme provides an alternative to traditional dementia education by connecting dietetic students to people with dementia and their carers in an out‐of‐placement setting.
- Contact with the families in the online environment led to positive learning outcomes about the lived experiences of dementia for the dietetic students.
- Students stated taking part in the online programme was better than not doing it, but acknowledged that a physical connection would have enhanced their experience.
- Narratives produced may support the design and delivery of future dementia education for dietetic and allied health professional students.

## 1 Introduction

There are approximately 55 million people living with dementia worldwide [1]. Current estimates suggest there are 944,000 people diagnosed with dementia in the United Kingdom, and this number is expected to continue rising [2]. Dementia is progressive, impacts cognition and leads to deterioration in functional abilities and often to behavioural disturbance [3].

People with dementia often require support from a range of healthcare professionals to treat and manage their symptoms, which can include swallowing difficulties, forgetting to eat and drink, resulting in malnutrition, a loss of independence during mealtimes, and changes in dietary habits [4]. Dietitians are essential in the provision of nutritional expertise and healthcare to people with dementia including assessment, care‐planning, education, and treatment [5]. In the United Kingdom, around 70% of registered dietitians in the NHS have direct patient contact [6] and most will likely have contact with people with dementia during their career. The rising prevalence of dementia is a recognised driver for change in the dietetic professional workforce development strategy [6], meaning that the future workforce needs to be equipped with the right skills, attitudes, and understanding during undergraduate education for them subsequently to provide high‐quality dietetic care to those with dementia [7, 8].

While there is a lack of research about dietetic students' knowledge and attitudes towards dementia, research more widely suggests that knowledge about ageing needs to be improved, and that their preference for working with older adults is low [9]. In a review of dietitians' knowledge and attitudes towards older adults in community and residential settings, negative assumptions and biases were reported [10]. Similarly, dietetic students have reported having negative perceptions of older adults, and barriers communicating and connecting with them [11]. These findings are concerning, as dementia predominantly impacts older adults and such perceptions are likely to impact care delivery.

To mitigate negative assumptions and biases towards older adults, initiatives to increase exposure, which are patient‐centred, and anti‐ageist should be considered [10]. Dietetic students who have had contact with older adults reported enhanced perception, understanding, and empathy towards ageing [11]; therefore, a similar approach might enhance awareness and insight about dementia during undergraduate education. Dementia education can be delivered in a variety of ways and wider research suggests that a combination of taught and interactive activities can enhance student understanding of dementia [7]. The key features associated with effective dementia education include: active participation; delivery by an experienced facilitator; group learning; sessions lasting 90 min and learning activities that can be applied in practice [12].

### 1.1 The Programme

Meeting these criteria, the Time for Dementia (TFD) programme was designed to improve knowledge and attitudes toward dementia in undergraduate healthcare students. Through longitudinal contact with families affected by dementia, students learn about the lived experiences of dementia [13]. TFD was designed and developed at Brighton and Sussex Medical School in partnership with the Alzheimer's Society and other participating universities in collaboration with people with dementia and their carers [14]. Families are recruited and supported to take part in the programme by the Alzheimer's Society. In the original TFD programme, student pairs completed in‐person home visits with a family (person with dementia and their carer) in the community over a period of 18–24 months. This longitudinal contact facilitates relational learning and has been shown to increase student understanding and empathy towards those with dementia [15]. Increases in knowledge of, and positive attitudes towards dementia have been reported in students who completed TFD when compared with students who did not complete the programme [16, 17]

The TFD programme was introduced to dietetic students at the University of Surrey in September 2020. However, local and national COVID‐19 restrictions came into force, meaning in‐person home visits could not occur. Responding to this, an online model was introduced to facilitate longitudinal contact between students and a family living with dementia. Using an online platform (e.g. Microsoft Teams [18] or Zoom [19]), groups of up to 12 students completed five online sessions over 18 months with a family living with dementia. Each online session was facilitated by a TFD facilitator. Throughout the programme, families were supported by the Alzheimer's Society TFD team. Each online session lasted 90 min. Students were provided with a learning guide before each session which contained suggested topics to explore with the family. Following each session, students met with a dietetic educator to reflect on their learning experiences. The programme aims for each group of students to meet with the same family throughout all five sessions. However, some families may withdraw from the programme, causing certain students to be re‐allocated to a different family. Upon completion of the programme, dietetic students completed a formative assessment, presenting their learning reflections to their cohort.

### 1.2 Aims

There is limited literature on dementia educational programmes for dietetic students, or online dementia education with experts by experience [20]. Therefore, the aim of this study was twofold. First, we aimed to understand the learning experiences of dietetic students in online TFD, and second, what impact the online delivery of the programme had on their learning experiences.

## 2 Materials and Methods

### 2.1 Study Design

To understand dietetic student perceptions of taking part in an online dementia educational programme, a qualitative study was completed that used focus group interviews to collect a rich data set that explored student experiences. An inductive, interpretive approach aligned with constructionism informed the relativist ontological and interpretivist epistemological stances adopted in this study [21, 22]. To address the aim, researchers sought to understand the varied narratives constructed by students following their participation in TFD, while also recognising their own influences on the interpretation of the findings. Reflexive thematic analysis was used as a flexible yet robust method to interpretatively generate key themes in these qualitative data that described student experiences [23, 24]. Focus groups were chosen as a pragmatic way to collect multiple student experiences while promoting the refinement of their ideas and perceptions of the programme through open discussion and debate. As there was limited time available to meet students, focus groups were a better fit as they were less time and labour intensive than other modalities such as individual interviews [25]. They also suited online data collection as participating students were located in a different part of the United Kingdom to the researchers [26]. These discussions were facilitated by an experienced qualitative researcher [Y.F.] to ensure the creation of a supportive and comfortable conversational space [27]. The focus groups were conducted at the end of the 2‐year programme.

### 2.2 Study Setting and Participants

Purposive sampling was used to recruit second‐year undergraduate dietetic students from two cohorts at the University of Surrey. Upon completion of TFD, the first cohort of students (*n* = 32) were invited to take part in a focus group in March 2022. The second cohort of students (*n* = 48) was approached in March 2023 following their completion of TFD, and they were also invited to take part in a focus group. No changes to the programme design nor delivery were made after the first cohort's focus group. Students from both cohorts were eligible to take part in the study as they had completed TFD.

### 2.3 Procedure

An email containing information about the study was sent to the students and a notice was placed on the students' digital learning platform inviting them to take part in the focus group. Students who expressed an interest in the study were sent a participant information sheet. Participation in this study was voluntary and informed written consent was obtained.

Focus groups comprising 3–7 students in each group were completed online using MS Teams by a qualitative researcher [Y.F./S.R.P.]. Each session lasted approximately 60 min, and was audio recorded using an encrypted Dictaphone. A topic guide was used to explore student experiences and learning outcomes in the programme. Topics for discussion included student perceived learning, educational needs, benefits and disadvantages of the programme, and the overall management of the programme. Data was collected, managed, and stored in line with local procedures and best practice. Audio files were transcribed verbatim and data were anonymised. Data was transcribed using a professional transcription service for the first cohort, and transcribed manually by researcher S.R.P. for the second cohort. Transcription files were password protected and access to these was restricted to authorised members of the research team.

### 2.4 Data Analysis

An inductive, thematic approach using Braun and Clarke's six steps was used to analyse the data [28]. Reflexive thematic analysis was chosen for its ability to produce high‐quality, reflexive accounts to understand and interpret qualitative data [23, 29]. S.R.P. led the analysis under the supervision of experienced qualitative researchers [Y.F./M.H.]. First, S.R.P. gained familiarity with the data by transcribing audio files, reading the transcribed files, and documenting initial ideas. Next, initial codes were generated by labelling sections of text. To enhance rigour, the research team independently hand‐coded the first focus group and created a framework of initial or open codes. A meeting was held to discuss the interpretation of the data and review any divergence in coding labelling or meaning. The remaining two transcripts were then coded by S.R.P. using NVivo 14 [30]. Codes were then sorted, organised, and combined into main themes and sub‐themes that captured the essence of the data. Themes were defined and labelled, and a report generated. Data sufficiency was deemed to be achieved as the researchers undertaking the analysis agreed that themes and sub‐themes adequately answered the aims of the study, working on the basis of the methodological framework of reflexive thematic analysis [31].

### 2.5 Rigour and Reflexivity

Field diaries were kept by researchers involved in the study. The research team met regularly to plan and reflect on the research journey and wider input was provided by dietetic educators EB and CJ to discuss the interpretation of the findings and seek feedback. The research team's use of a reflective approach was necessary to ensure rigour throughout all stages of the analysis. This approach allowed the team to discuss and refine ideas that developed during the analysis to form a more robust interpretation and presentation of the data. The research team involved in data collection and analysis had a variety of backgrounds and interests in dementia. Researcher S.R.P. is a final‐year medical student at Brighton and Sussex Medical School. He has a special interest in dementia and dementia education and has been involved with TFD as a student representative during medical school. Researcher Y.F. is an adult nurse and she supports the delivery of TFD in participating universities. M.H. is a dementia researcher, and she supports the evaluation of TFD. The team discussed their positionality regularly to consider their own influences during data collection, and during the interpretation and construction of findings.

### 2.6 Ethics

Ethical approval was obtained from the NHS Health Research Authority, London Queen Square Research Ethics Committee (15/LO/0046). Researchers involved in the recruitment and facilitation of the focus groups were not previously known to the students. Participation in the research was voluntary, and students did not receive reimbursement for their time.

While no ethical concerns were disclosed during the focus groups, procedures were in place to support researchers to manage potential issues. These included regular reflective research discussions, training on ethical considerations, and clear reporting avenues in line with university ethical protocols.

## 3 Results

Fourteen dietetic students took part in three focus groups. There were three students in group one, four in group two, and seven students in focus group three. Two focus groups were conducted with participants from the first cohort, and another focus group was later conducted with participants from the second cohort. Student demographics are presented in Table 1.

**Table 1:** Demographics of participating dietetic students.

Demographic	Number	Mean	SD	Range
Age		28	9.5	20–52
Sex				
Female	12			
Male	2			
Ethnicity				
White British/European	13			
Asian/Asian British	1			
Experience of dementia before programme				
Yes	6			
No	8			
Details of experience				
Family/friend	4			
Work experiences	0			
Both	2			
Participating programme year				
2020–2022	7			
2021–2023	7			

Three main themes were generated from the data: (i) *gaining a holistic understanding of the experience of life with dementia,* (ii) *shaping future practice, adapting approaches for dementia care*, and (iii) *optimising learning in an online environment*. Themes and sub‐themes are discussed in detail next. Table 2 outlines the themes and sub‐themes and provides examples of their content.

**Table 2:** Overview of themes and sub‐themes generated from focus groups.

Theme	Subtheme	Content
Gaining a holistic understanding of the experience of life with dementia	Understanding the shared experience of dementia Challenging misconceptions and stigma Understanding health and support systems	A broad understanding of the lived experiences of dementia, and life as a carer Students preconceived perceptions of dementia were challenged, reframing negative stereotypes Broader understanding of the availability and limitations of health and support systems for dementia, an understanding of families' negative encounters in healthcare settings
Shaping future practice, adapting approaches for dementia care	Intentions to adapt a person‐centred care approach Adapting communication for people with dementia A need for change, raising the profile of the dieticians role	Seeing the person behind dementia and the carer, insight into emotional experiences, the importance of person‐centredness, practical and helpful strategies around diet and nutrition in dementia Learning to communicate with a person with dementia, helpful communication strategies, principles of good communication, building rapport, trust, and confidence to explore sensitive topics Limitations in current care practices, importance of routine health monitoring, a lack of family awareness about the role of the dietitian, need to raise the profile of dietetics in primary care
Optimising learning in an online environment	The challenges establishing personal connections online Guided support enhances learning experiences Seeking a broader understanding of dementia	Positive experiences connecting with families online, enhanced accessibility, dynamics of the online group discussions, challenges building rapport online Well‐organised sessions, helpful resources, trained facilitator prompted discussions and accountability The need for taught dementia education, integration of dietetic topics into sessions, more contact with more families with dementia

## 4 Gaining a Holistic Understanding of the Experiences of Life With Dementia

Through online contact, students gained a holistic understanding of the experiences of life with dementia. Shared discussions resulted in a broader understanding of dementia that extended beyond the person living with the condition. This was evident in the students' rich descriptions and new insight about the impact of dementia on the family experience, a greater awareness around misconceptions towards dementia, and a realisation that health and support systems are often unfit for the needs of people with dementia. Three subthemes informed their holistic understanding of dementia: (a) understanding the shared experience of dementia, (b) challenging stigma and misconceptions and (c) understanding the health and support systems.

### 4.1 Understanding the Shared Experience of Dementia

Through longitudinal interactions with a family online, students gained deeper insight into daily life and the issues that families with dementia can experience day to day. For example, some students highlighted challenges families experienced accessing healthcare, while others discussed the negative impact that COVID‐19 restrictions had on the socialisation skills of the person with dementia. Gaining the carer's perspective was described as a valuable learning experience. Students described increased understanding about the role of the carer and the impact of dementia on relationships in the family.

A realisation occurred that dementia not only affected the person living with the condition, but also impacted the wider family. Sometimes, students encountered situations when the person with dementia and the carer held differing perceptions about dementia. This was viewed as a challenge to having an open conversation. For example, when a person with dementia did not fully accept their condition, but their carer was more open to embracing it, students suggested that certain topics could not always be freely discussed in the presence of each other. Nevertheless, a common point raised by the students was that both people with dementia and their carers found it difficult to talk about the future and how they would cope when the condition progressed further.

…he just didn't want to discuss the negative side of what could happen with him in the future….

(Focus group 1, Student 3)

### 4.2 Challenging Misconceptions and Stigma

Students suggested that misconceptions and stereotypes are commonly associated with dementia. Some students suggested that dementia is often viewed as a ‘person‐defining disease’ or a ‘life sentence’ in society but by spending time with the family, students were able to confront their own preconceived ideas about dementia, leading to more positive views and a recognition that people with dementia should be treated fairly.

…If you live with dementia, it's the same as everyone else… they [People with dementia] want to be treated like just normal people and not their condition, which was a big reflecting moment for me….

(Focus group 3, Student 1)

By interacting with the family, students recognised the individuality of the diagnosis and the diversity of coping strategies taken by the person with dementia. It was suggested by some that the progression of dementia was less pronounced than they expected, which helped them reframe their assumptions that dementia progresses quickly towards the later stages. Some students met more than one family and suggested that contrasting experiences helped them to gain a greater understanding of the diversity of experiences of dementia.

### 4.3 Understanding Health and Support Systems

There was an impact on students' understanding of the health and associated support systems that are available for families affected by dementia. Students described these health and support systems as deficient and unsuitable to support the family's needs. Many students were surprised by the persistent difficulties that the family faced when navigating and accessing support systems. For example, routine health monitoring appointments were seen as inconsistent, and there was a perceived lack of dietitian support available for families when it was required.

Students described negative interactions that families experienced when interacting with healthcare professionals. Here, students discussed families' experiences of care that were viewed as lacking in person‐centred approaches, care encounters that focused on clinical presentation and diagnosis rather than seeing the person, and family accounts of the person with dementia being overlooked. Students reflected on these accounts and perceived them as examples of poor practice.

…We all reflected on it… it kind of made us really sad that he had such a bad experience with the healthcare professionals that we all just didn't want to be that kind of healthcare professional when we qualify…

(Focus group 2, Student 1)

## 5 Shaping Future Practice, Adapting Approaches for Dementia Care

The second theme describes how their experience of TFD motivated student intentions to adapt their approaches towards people with dementia during future practice. The theme describes a growing awareness that change in health and care systems is needed to provide effective dementia care. Better awareness of the importance of person‐centred care, the need to adapt their communication, and the need to raise the profile of the role of the dietitian in the community were discussed.

### 5.1 Intentions to Adapt a Person‐Centred Care Approach

The principles of person‐centred care were described as fundamental for future practice for dietetic students. Students stated that TFD gave them an opportunity to understand the person with dementia and their carer outside of the clinical setting. Gaining insight into the family's home life, including the coping strategies that they had put in place, was described as a valuable learning outcome. Students highlighted the importance of understanding the emotional impact that a dementia diagnosis has on the family. Recognising the importance of person‐centred approaches, some students remarked that advice given during consultations with healthcare professionals may sometimes be impractical for the person with dementia. For example, when the clinician does not have a holistic understanding of that person and provides advice that is inadequate for that individual's circumstances.

Students described hearing about useful practical strategies from their TFD families, such as keeping weekly planners or planning out meals, and considered utilising this knowledge by sharing these strategies with their future patients. Similarly, students reflected that nutritional strategies need to be implemented differently depending on the stage of dementia and the nutritional and other support needs of the person with dementia. Students agreed that person‐centred care is a simple and necessary approach, they aimed to implement going forward in their careers as clinicians.

…I think you can like tailor your advice to suit them, because they're more likely to make changes then if you have took their needs into account. …

(Focus group 1, Student 2)

### 5.2 Adapting Communication for People With Dementia

The ability to effectively communicate with people with dementia and their carers was described as an important learning outcome for students. Strategies that enhanced communication were described, such as the need for flexibility with the person with dementia by allowing them extra time to process information. The importance of addressing both the person with dementia and the carer throughout the conversation to prevent excluding any person was highlighted, and attention to body language was identified as a key requirement in the establishment of good rapport.

The ability to establish good rapport and trust with the family was viewed as an important facilitator of student learning. By creating a trusting environment, students suggested that they could address sensitive topics of conversation. Some students explored the impact of dementia on the family's financial situation, while other students discussed episodes when the family had encountered stigma or judgement from healthcare professionals. Students agreed they gained confidence when discussing emotional topics with their TFD families and appreciated this as a skill to take forward into their future practice.

…Being able to see those types of relationships and ask all the difficult questions is… gonna make it easier in the future to be able to address the carers and the patient at the same time…

(Focus group 3, Student 1)

### 5.3 A Need for Change, Raising the Profile of the Dieticians Role

All students discussed the need to improve current care practices. Students suggested that routine health monitoring could be beneficial in the management of dementia and could aid preventative strategies. Students suggested that regular nutritional reviews, and blood pressure checks might highlight early warning concerns in dementia. Students also suggested that this could be built into their own practice. By routinely completing basic health checks, students considered that better outcomes could be achieved for the person with dementia, the carer, and the NHS.

Students suggested that greater public awareness about the role of dietitians is needed across primary care settings. Most of their TFD families were unaware of the role of dietitians in healthcare, or the reasons why a person with dementia might need the support of a dietitian. Students suggested that families with dementia should have awareness about how to access dietetic care, what services are available in their area, and how a dietitian can help them should any problems arise. Similarly, raising awareness about the role of the dietitian in dementia to other primary care professionals was viewed as an important facilitator of better care. To provide holistic care to people with dementia, some students suggested a need for greater integration of dietetic care into healthcare practices and input in multi‐disciplinary meetings.

I think probably first line healthcare professionals… I think maybe more education regarding dietetic care could be arranged for them, because… [when they] see that the patient is not eating very well, or have difficulty swallowing maybe it just sparks in their mind [that] this person may need dietetic care…

(Focus group 2, Student 4)

## 6 Optimising Learning In an Online Environment

This theme describes student experiences of the online learning environment. Overall, the TFD programme was described as a positive opportunity to learn about dementia by interacting with families living with the condition. While the addition of learning resources and the presence of a facilitator at each session enhanced their experiences, students discussed challenges in establishing and maintaining interpersonal connections in the online environment. There were limitations in the scope of content covered that suggested a need to expand dementia education for practical and discipline‐specific relevance.

### 6.1 The Challenges Establishing Personal Connections Online

There were facilitators and barriers associated with the online delivery of TFD. Overall, TFD was viewed positively, and was described by students as an enjoyable and interesting learning experience. The ability to connect with families online was described as an accessible option meaning students were able to connect with families outside their local area.

However, there were challenges identified by students when conversing with a family in an online environment. Due to the number of students allocated to each online session, the conversation was sometimes viewed as fragmented. Many students described the need to wait their turn to contribute, meaning that by the time a student could participate, the conversation had already moved on, and students had to change their planned question for the family. Additionally, some students found it difficult to build rapport and ask personal questions due to the nature of the online environment. However, most students agreed that taking part in the online programme was better than not doing it at all, but acknowledged that a physical connection would have enhanced their experience.

…being able to do it online… would make it, you know, one hundred times better than not doing anything at all…

(Focus group 1, Student 4)

### 6.2 Guided Support Enhances Learning Experiences

Overall, students had clear understanding about the learning expectations associated with TFD. Preparatory materials provided before online sessions were described as helpful. For example, students described using the learning guides to prepare questions ahead of their sessions. While the learning guides enhanced preparedness, some students also suggested that conversations become repetitive over time or that sensitive questions related to life in the future with dementia were difficult to discuss due to students' fear of unsettling the TFD family.

The presence of a group facilitator from the Alzheimer's Society was identified as essential during the sessions. Students described feeling reassured that the facilitator could manage situations if difficult topics were raised, thereby creating a safe discussion environment. Facilitators enhanced students' accountability, encouraged questions, contributed to the discussion, managed time between students, and prompted all students to interact with families.

it was obviously useful having the Facilitator in the session to kind of set out the ground rules and sort of when we were typing questions in the chat

(Focus group 1, Student 1)

### 6.3 Seeking a Broader Understanding of Dementia

During the focus group discussions, students suggested changes to improve the future delivery of the programme. Many students suggested that teaching sessions about dementia and its pathophysiology would be beneficial to their learning before commencing TFD and would help prepare them more adequately for their first session. Integration of dietetic topics into the sessions was raised as a point to further develop the sessions. Several students highlighted that their TFD families had never been in contact with a dietitian, making the students feel they did not gain as much as they could have done when discussing dietary‐specific topics. To tackle this issue, some students suggested the inclusion of sessions with different TFD families, specifically those that might be in the later stages of dementia or might currently require dietetic support. In this way, the students remarked they could have gained a broader understanding of the impact of dietary care in people with dementia. The students similarly emphasised how hearing from previous students who participated in the programme would have helped them better understand why it is important and useful to engage with it.

In the first few visits [online sessions] I was just like… ‘Why am I doing this?’ And then obviously placement happened and if I'd heard somebody [say] ‘You're going to come into a lot of contact with dementia patients in the hospital’… I think I would have understood the concept more in the beginning

(Focus group 3, Student 6)

## 7 Discussion

This study aimed to understand the experiences of dietetic students taking part in a longitudinal, online dementia educational programme. Three main themes shaped student experiences. These were (i) *gaining a holistic understanding of the experience of life with dementia,* (ii) *shaping future practice, adapting approaches for dementia care* and (iii) *optimising learning in an online environment*.

As dementia becomes more common in clinical populations and in the community, most healthcare students during their future careers will work with people with dementia. Therefore, the healthcare workforce needs the right skills and attitudes to provide high‐quality care for people with dementia. Previous literature suggests that educational programmes facilitating in‐person contact have improved knowledge, attitudes, and understanding towards dementia in healthcare students [17, 32, 33]. In this study, contact between dietetic students and families with dementia was facilitated in an online environment, for which outcomes have not been previously explored. The results suggest that positive self‐reported outcomes, similar to previous findings are observed: students gained a deeper understanding of the lived experiences of dementia and recognised the importance of person‐centred approaches. These positive relational learning experiences appear to contribute to changes in misconceptions and stigma towards dementia. This suggests programmes such as TFD in an online format might contribute to improvements in dementia education.

However, the development of rapport between students and families during sessions is a fundamental aspect of the TFD programme and limitations in establishing and building relationships were highlighted due to the online format. Previous research has suggested that continuous interaction between students and families can result in enhanced trust and increased willingness to discuss experiences honestly [15]. However, some barriers were identified here in the establishment of these relationships in the online setting. The ability to actively, rather than passively engage in the learning process can enhance learning [34], and the findings here suggest that active engagement might be challenged in larger group settings in the online environment. When a larger number of individuals were allocated to an online session, free‐flowing conversation was challenged. Students needed to wait their turn, and the conversation was described as fragmented. This may suggest that passive approaches to learning also occurred, where students needed to wait longer periods of time before they had an opportunity to connect with and engage with the family. To ensure meaningful interactions between the students and their teachers, Taft et al. [35] suggest that online classes should not exceed 20 students. To build rapport and achieve fruitful conversations, our findings align with wider literature that suggests online groups should be limited to 6–12 students [36, 37]. By reducing group sizes, students in online learning environments may have more opportunities to engage in active conversation.

In the online environment, some students avoided discussions that centred on sensitive topics due to fear of upsetting the family. This could be a limitation for students' learning. Programmes like TFD aim to enhance understanding of dementia through longitudinal rapport, meaning students gain insight into the challenging experiences of living with dementia. While communication training can help healthcare students feel more confident when interacting with people with dementia [38], and increased exposure to dementia in education settings can increase empathy and reduce anxiety when interacting with people with dementia [39], challenges establishing rapport in the online environment might impact student ability to explore sensitive topics. Additionally, one distinction between face‐to‐face and online contact is the opportunity for people with dementia and carers to meet individually with students and have separate conversations. Future research should investigate how student confidence and family willingness to discuss sensitive or emotive topics are affected when using online communication platforms.

This study adds to the limited literature exploring undergraduate dementia education in dietetics and has some specific implications for this speciality. The key learning outcomes identified in this study align with the British Curriculum Framework for the pre‐registration education and training of dietitians [40]. For example, students gained an understanding of person‐centred approaches, they highlighted awareness of the need to adapt communication, and they recognised the importance of multidisciplinary teamwork. They also described inequalities in healthcare in dementia, and there was a heightened awareness of the role of dietitians in the community and that integration of dietitians in primary care settings needs to be improved, which reflects the current understanding of services [41]. Findings suggest that the programme can increase a positive framing of dementia care. Improving attitudes towards people with dementia needs to be a priority in dietetic undergraduate programmes. A study of dietetic students found that career preferences for working with older adults were low across the United Kingdom [9] and this aligns with wider research on healthcare student preferences for working with people with dementia [42].

To enhance dietetic learning, findings suggest that students could be better introduced to the context of dietetics in dementia care before starting TFD, and sessions could be scaffolded around dietetic‐specific content. This suggests that TFD, which runs over nine different healthcare programmes, could be further tailored to include profession‐specific learning objectives. There may also be a value in introducing interprofessional learning objectives. The role of dietitians in the primary care multidisciplinary team has been well established, especially to help people living with dementia experiencing weight loss and/or changes in taste and appetite [43], and was recognised by students in this study. A possible contribution to building skilled teams could lie in multidisciplinary dementia educational interventions involving students from different healthcare courses. A review of the literature has already suggested that the integration of interprofessional education, with long‐term experiential programmes, may encourage students to have a multidisciplinary approach in future practice [44]. Therefore, it would be of interest to study the impact of an interprofessional version of TFD.

## 8 Strengths and Limitations

There is limited literature available on dementia education for dietetic students; therefore, this study contributes to that body of knowledge. The qualitative nature of the study is one of its primary strengths as it allowed a deeper and more meaningful understanding of student experiences of the TFD programme and its impacts. In the online environment, focus groups facilitated rich discussion, which included a range of student perspectives and reflections on their learning experiences. The outcomes from the study are transferable as the findings can inform the iterative development of the TFD programme to improve its wider roll‐out. In addition, findings are relevant for educators designing similar online educational programmes. By understanding their learning experiences in this study, educators can consider strategies to manage challenges and facilitators to improve online educational delivery.

However, the study also has some limitations. Students were recruited from one discipline and one University. Students from a range of healthcare disciplines and other universities might have different experiences and outcomes that could provide further insight into student perceptions of online delivery. The use of focus groups may have limited some students from discussing their experiences more openly. Disparities in group sizes across the three focus groups also could have affected the ability of students to speak within larger groups or to speak freely in smaller groups. Furthermore, students interviewed only had an experience of online delivery that was confounded with the experience of COVID‐19 pandemic at a time when all education had been disrupted. Additionally, data from focus groups were collected at a single time point. Using repeated focus groups pre‐ and post‐TFD might have generated greater insight about the impact of the programme on student learning outcomes. Future work should investigate the differences between in‐person and online delivery of TFD in a direct comparison.

## 9 Conclusion

This study is among the first to explore the experiences of dietetic students in online dementia education. Ensuring that the future workforce has appropriate knowledge, understanding, and attitudes to provide high‐quality care to people with dementia needs to be a priority in undergraduate healthcare education. Positive learning outcomes reported in this study suggest that dementia education, provided in the online environment, can successfully enhance holistic knowledge and motivate intentions to improve future practice in dietetic students. However, in future iterations of this model, or in the design and delivery of similar online programmes, educators should consider the interpersonal barriers experienced by students that may limit some aspects of their learning.

Future research should explore how longitudinal relationships can be established and maintained between students and people living with dementia in the online setting and investigate differences in outcomes from in‐person visits. Research on the response of the face‐to‐face version of TFD on dietetic students would be of interest.

## Author Contributions

The study design was conceptualised by Stephanie Daley, Cecile Jones, Emily Norris, and Yvonne Feeney. Data was collected by Yvonne Feeney and Santiago Rodriguez Pena, and analysis completed by Yvonne Feeney, Santiago Rodriguez Pena, and Molly Hebditch. All authors contributed to the interpretation of findings and the production of the manuscript.

## Ethics Statement

Ethical approval was obtained from the NHS Health Research Authority London Queen Square Research Ethics Committee (15/LO/0046).

## Conflicts of Interest

The authors declare no conflicts of interest.

### Peer Review

The peer review history for this article is available at https://www.webofscience.com/api/gateway/wos/peer-review/10.1111/jhn.70069.

## Transparency Declaration

The lead author affirms that this manuscript is an honest, accurate, and transparent account of the study being reported. The reporting of this study is compliant with the consolidated criteria for reporting qualitative research. The lead author affirms that no important aspects of the study have been omitted.

## Data Availability

The data that support the findings of this study are available from the corresponding author upon reasonable request.
